# Bibliometric analysis of academic journal recommendations and requirements for surgical and anesthesiologic adverse events reporting

**DOI:** 10.1097/JS9.0000000000000323

**Published:** 2023-05-03

**Authors:** Tamir N. Sholklapper, Jorge Ballon, Aref S. Sayegh, Anibal La Riva, Laura C. Perez, Sherry Huang, Michael Eppler, Gregg Nelson, Giovanni Marchegiani, Robert Hinchliffe, Luca Gordini, Marc Furrer, Michael J. Brenner, Salome Dell-Kuster, Chandra Shekhar Biyani, Nader Francis, Haytham M.A. Kaafarani, Matthias Siepe, Des Winter, Julie A. Sosa, Francesco Bandello, Robert Siemens, Jochen Walz, Alberto Briganti, Christian Gratzke, Andre L. Abreu, Mihir M. Desai, Rene Sotelo, Riaz Agha, Keith D. Lillemoe, Steven Wexner, Gary S. Collins, Inderbir Gill, Giovanni E. Cacciamani

**Affiliations:** a Catherine and Joseph Aresty Department of Urology, Keck School of Medicine, Los Angeles, California; b Department of Urology, Einstein Healthcare Network, Philadelphia, Pennsylvania; c Department of General Surgery, Digestive Disease & Surgery Institute, Cleveland Clinic Foundation, Cleveland, Ohio; d Department of Surgery, Johns Hopkins Medicine, Baltimore, Maryland; e Department of Otolaryngology-Head & Neck Surgery, University of Michigan Medical School, Ann Arbor, Michigan; f Trauma Department, Emergency Surgery and Surgical Critical Care; g Department of Surgery, Massachusetts General Hospital and the Harvard Medical School, Boston, MA, USA; h Department of Surgery, University of California San Francisco (UCSF), San Francisco, California; i Department of Colorectal Surgery, Cleveland Clinic Florida, Weston, Florida, USA; j Department of Urology, Royal Melbourne Hospital, Parkville, Victoria, Australia; k Department of Urology, Guy’s and St Thomas’ NHS Foundation Trust, London; l Department of Vascular Surgery, University of Bristol, Bristol; m Harley Clinic, London; n Department of Urology, Leeds Teaching Hospitals NHS Trust, Leeds; o Department of General Surgery, Yeovil District Hospital NHS Foundation Trust, Yeovil; p UK EQUATOR Centre, Centre for Statistics in Medicine, Nuffield Department of Orthopaedics, Rheumatology, & Musculoskeletal Sciences, Botnar Research Centre, University of Oxford, Oxford, UK; q Center for Colorectal Disease, St Vincent’s University Hospital, Dublin, Ireland; r Department of Cardiac Surgery, Cardiovascular Center, Inselspital, Bern; s Department of Urology, University of Bern, Inselspital, Bern; t Clinic for Anaesthesia, Intermediate Care, Prehospital Emergency Medicine and Pain Therapy; University Hospital Basel, Switzerland; u Department of Urology, Ludwig-Maximilians-University Munich, Munich, Germany; v Department of Urology, Intitut Paoli-Calmettes Cancer Centre, Marseille, France; w Division of Oncology, Unit of Urology, URI, IRCCS Ospedale San Raffaele; x University Vita-Salute San Raffaele, Milan; y Division of Endocrine Surgery, “Agostino Gemelli” School of Medicine, University Foundation Polyclinic, Catholic University of the Sacred Heart, Rome; z Department of General and Pancreatic Surgery, University of Verona, Verona; aa Department of Ophthalmology, University Vita-Salute, IRCCS Ospedale San Raffaele, Milan, Italy; bb Department of Obstetrics & Gynecology, Cumming School of Medicine, University of Calgary, Calgary, Alberta; cc Department of Urology, Queen’s University, Kingston, Ontario, Canada

**Keywords:** adverse event reporting, quality improvement, surgical safety

## Abstract

Standards for reporting surgical adverse events (AEs) vary widely within the scientific literature. Failure to adequately capture AEs hinders efforts to measure the safety of healthcare delivery and improve the quality of care. The aim of the present study is to assess the prevalence and typology of perioperative AE reporting guidelines among surgery and anesthesiology journals.

**Materials and methods:**

In November 2021, three independent reviewers queried journal lists from the SCImago Journal & Country Rank (SJR) portal (www.scimagojr.com), a bibliometric indicator database for surgery and anesthesiology academic journals. Journal characteristics were summarized using SCImago, a bibliometric indicator database extracted from Scopus journal data. Quartile 1 (Q1) was considered the top quartile and Q4 bottom quartile based on the journal impact factor. Journal author guidelines were collected to determine whether AE reporting recommendations were included and, if so, the preferred reporting procedures.

**Results:**

Of 1409 journals queried, 655 (46.5%) recommended surgical AE reporting. Journals most likely to recommend AE reporting were: by category surgery (59.1%), urology (53.3%), and anesthesia (52.3%); in top SJR quartiles (i.e. more influential); by region, based in Western Europe (49.8%), North America (49.3%), and the Middle East (48.3%).

**Conclusions:**

Surgery and anesthesiology journals do not consistently require or provide recommendations on perioperative AE reporting. Journal guidelines regarding AE reporting should be standardized and are needed to improve the quality of surgical AE reporting with the ultimate goal of improving patient morbidity and mortality.

## Introduction

Highlights

- This bibliometric analysis investigated journal adverse event (AE) reporting guidelines.
- Only 46.5% of the journals studied included any mention of surgical AE reporting.
- AE reporting recommendations varied significantly by geographic region.
- General surgery, urology, and anesthesia are most often recommended for AE reporting.

Surgical adverse events (AEs) are known to have a significant impact on patients resulting in declining quality of life and level of physical and mental health^1^. Further, AEs can be costly to patients and the healthcare system^2^, and their subsequent management had a significant financial impact on the healthcare system and has been found to be associated with a 119% increase in the cost of care^3^. Understandably, measuring the quality of healthcare delivery to ensure patient safety is an area of growing interest for clinicians, policymakers, payers, and the public. While these quality metrics can be based on structure, process, or outcomes, they are most often based on outcomes alone, underscoring the value of ensuring standardized and reproducible outcome data reporting^4^–^6^. Further, these performance metrics frequently inform hospital training initiatives and impact insurance reimbursement and, therefore, impact profit margins with a potential influence on the value of care with implications for financial sustainability or healthcare organizations.

An essential component of improving procedural and surgical outcomes is identifying and reducing perioperative AEs or other negative outcomes of procedures. However, AE reporting across surgeries is highly variable, underscoring the need for guidelines for standardized reporting https://journals.lww.com/annalsofsurgery/Abstract/9900/Severity_Grading_Systems_for_Intraoperative.445.aspx 7,8. Choosing the appropriate, rigorous set of AE reporting guidelines is also critical for accurate measurement and avoiding the pitfall of underestimating these events^9^,^10^. Attempts to standardize AE reporting in the surgical and anesthesiology literature have had encouraging results^11^,^12^. Several studies have utilized postoperative complication reporting guidelines to assess perioperative AEs, whereas other studies have evaluated reporting habits^13^–^17^. Despite these efforts, perioperative AEs remain underreported, and a significant portion of recent publications do not adequately report AEs in a standardized fashion^17^. One study separately examined intraoperative complications alongside postoperative complications in surgical trials and found that they are often bundled together, improperly defined, or simply not reported^8^. Specifically, of the 46 trials included in the aforementioned study, intraoperative and postoperative complications were reported separately in 42% and together in 15%^8^. Indeed, journal author guidelines have a vital role in that they normalize requirements for submission (i.e. journals should offer guidelines not only on how an article should be formatted but also on the requisite standardization for critically appraise and theoretical study replication).

The aim of the present bibliometric analysis was to assess the prevalence and typology of perioperative AEs reporting requirements and recommendations among journals across surgical subspecialties and anesthesiology. We hypothesized that the majority of journals require or suggest the use of standardized AE reporting guidelines.

## Materials and methods

### Data acquisition

The list of journals was aggregated by searching the 2020 SCImago Journal Rankings (SJR) by category (i.e. specialty). The SCImago Journal & Country Rank portal (www.scimagojr.com) is a bibliometric indicator database based on data from Scopus. Categories captured included: general surgery, transplant surgery, obstetrics and gynecology (OB-GYN), urology, otorhinolaryngology (ear, nose, and throat surgery [ENT]), orthopedic surgery, emergency medicine, ophthalmology, and anesthesia. In cases where journals were listed under multiple categories, they were included as separate entries to account for differences in journal influence by specialty. Characteristics captured from SCImago included SCImago Journal Ranking, SJR quartiles, H-index, document counts, citation counts, country, region, publisher, and category.

Next, the official website for each journal was manually searched for author instructions. In November 2021, three of the study group (A.S.S., A.L.R., and L.C.P.), after proper training regarding the data to extract, collected the outcome of interest from the list of journals retrieved in SCImago into a database under the supervision of a senior author (G.E.C.). The training was intended to familiarize the extractors with the topic and methods and the data collection sheet and solve concerns that could occur during data extraction. It consisted of teaching sessions where the senior author explained the meaning of each of the variables to collect and where to retrieve them from the ‘author’s guidelines’ webpage. The data collected included any general or specific recommendation or reference to reporting of surgical AEs and collected the data of interest into a database. General recommendations included any mention of complication reporting; reference to any guidelines listed in the comprehensive database of reporting guidelines known as the EQUATOR (Enhancing the QUAlity and Transparency Of health Research) Network library (www.equator-network.org); or reference to any generic AEs reporting guidelines. Specific recommendations included reference to criteria for capturing or grading surgical AEs. These recommendations were further subdivided into intraoperative and postoperative AEs reporting recommendations.

### Statistical analysis

Each journal category was evaluated for the number and percent of journals that recommended or provided some guidance on procedural AE reporting. These numbers were subgrouped by SCImago ranking, quartile, region, and country. Percent of journals recommending specific surgical AE reporting or classification was similarly determined by category.

A multivariable logistic regression model was fit to evaluate the role of the journal quartile, region of the editorial office, and category on the odds of any AE reporting recommendation^18^. Tests were two-tailed, and a *P* value less than 0.05 was considered significant. Analysis was conducted using JMP Pro version 16.0.0 (2021 SAS Institute Inc, Cary, North Carolina, USA).

## Results

**Figure 1:**
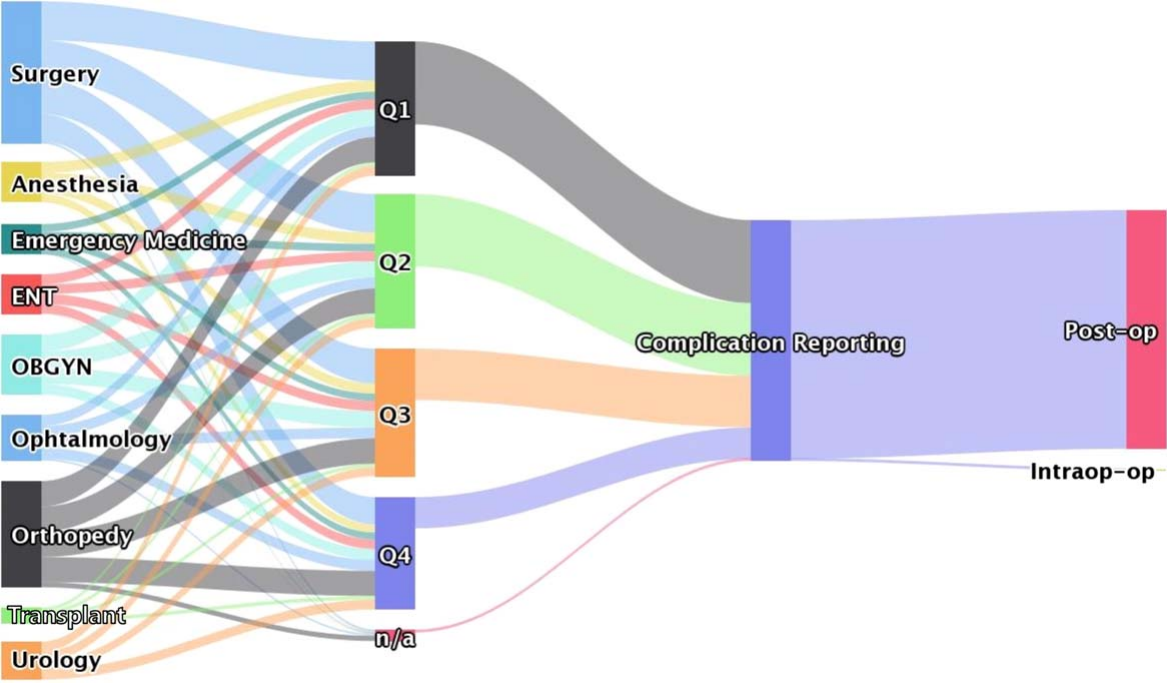
Characteristics of journals reporting the recommendations and guidelines on how to report the perioperative adverse events. The width is proportional to the quantity represented. ENT, ear, nose, and throat; Intra-op, intraoperative; OB-GYN, obstetrics and gynecology; Q, quartile.

In total, 1409 journals were identified, of which 655 (46.5%) recommended some form of AE reporting and 754 (53.5%) did not (Table 1). Among the SJR categories, general surgery, urology, and anesthesia journals had the greatest proportion recommending AE reporting (59.1, 53.3, and 52.3%, respectively). Transplant surgery had the lowest proportion at 26.8%. The proportion recommending AE reporting decreased in the order of SJR quartile from 61.8% in the first quartile (Q1) to 27.9% in Q4 (Fig. 1). Journals based in Western Europe, North America, and the Middle East had the greatest proportion recommending AE reporting (49.8, 49.3, and 48.3, respectively). Journals in Eastern Europe had the lowest rate at 9.6%. Countries with the greatest proportion of journals recommending surgical AE reporting were New Zealand, Switzerland, and India (64.3, 62.5, and 60.4, respectively). Additional characteristics of these journals, including the H-index and recommendations by the publisher, are available in Appendix Table A.1, Supplemental Digital Content 1, http://links.lww.com/JS9/A418.

**Table 1:** Summary of journal surgical adverse event reporting guidelines by journal category, SJR quartile, region, and country.

	Provided some guidance on reporting adverse events, *N* (%)	Provided no guidance on reporting adverse events, *N* (%)
Overall	655 (46.5)	754 (53.5)
SJR categories		
General surgery	228 (59.1)	158 (40.9)
Transplant surgery	11 (26.8)	30 (73.2)
OB-GYN	75 (45.7)	89 (54.3)
Urology	56 (53.3)	49 (46.7)
ENT	42 (39.3)	65 (60.7)
Orthopedic surgery	102 (35.4)	186 (64.6)
Anesthesia	58 (52.3)	53 (47.7)
Emergency medicine	36 (43.4)	47 (56.6)
Ophthalmology	47 (37.9)	77 (62.1)
SJR quartile		
Q1	225 (61.8)	139 (38.2)
Q2	197 (54.4)	165 (45.6)
Q3	140 (40.5)	206 (59.5)
Q4	85 (27.9)	220 (72.1)
Region		
Western Europe	315 (49.8)	318 (50.2)
Northern America	208 (49.3)	214 (50.7)
Asiatic Region	65 (43.9)	83 (56.1)
Eastern Europe	7 (9.6)	66 (90.4)
Middle East	29 (48.3)	31 (51.7)
Others	30 (41.7)	42 (58.3)
Country		
Australia	4 (44.4)	5 (55.6)
Austria	2 (33.3)	4 (66.7)
Brazil	6 (33.3)	12 (66.7)
Canada	2 (33.3)	4 (66.7)
China	5 (26.3)	14 (73.7)
Egypt	6 (60.0)	4 (40.0)
France	15 (46.9)	17 (53.1)
Germany	37 (48.1)	40 (51.9)
India	29 (60.4)	19 (39.6)
Iran	10 (47.6)	11 (52.4)
Italy	23 (54.8)	19 (45.2)
Japan	8 (25.0)	24 (75.0)
Mexico	1 (20.0)	4 (80.0)
Netherlands	44 (55.7)	35 (44.3)
New Zealand	9 (64.3)	5 (35.7)
Poland	0 (0.0)	21 (100.0)
Russia	3 (13.0)	20 (87.0)
Singapore	2 (33.3)	4 (66.7)
South Korea	18 (56.3)	14 (43.8)
Spain	14 (43.8)	18 (56.3)
Switzerland	20 (62.5)	12 (37.5)
Turkey	16 (48.5)	17 (51.5)
United Kingdom	149 (48.2)	160 (51.8)
United States	206 (49.5)	210 (50.5)

*P* values for *χ* ^2^ analyses.

ENT indicates ear, nose, and throat; OB-GYN, obstetrics and gynecology; Q, quartile; SJR, SCImago journal rank.

The percentage of journals with surgical AE reporting recommendations and requirements were collected and are reported by category in Table 2. In addition, journals recommending/requiring reporting guidelines via the EQUATOR Network website were delineated. Overall, 24.7% of journals recommended guidelines listed in the EQUATOR Network library, of which the top three were in anesthesia (36.0%), general surgery (29.5%), and OB-GYN (29.3%) journals. Further descriptions of EQUATOR Network guidelines and other guidelines are shown in Appendix Table A.2, Supplemental Digital Content 1, http://links.lww.com/JS9/A418. Few journals recommended specific guidelines or described their own guidelines for reporting intraoperative or postoperative AEs. Overall, only 6.3% of all journals captured in our study included specific recommendations, of which urology, general surgery, and orthopedic surgery journals had the greatest proportion of journals with these recommendations at 11.4, 11.1, and 6.6%, respectively. Examples of specific references are included in Appendix Table A.3a and A.3b, Supplemental Digital Content 1, http://links.lww.com/JS9/A418 for postoperative and intraoperative AEs.

**Table 2:** Endorsement of complication reporting with general and specific recommendations.

	Overall	General surgery	Transplant surgery	OBG-YN	Urology	ENT	Orthopedic surgery	Anesthesia	Emergency medicine	Ophthalmology
*n* of journal	1409	386	41	164	105	107	288	111	83	124
Any recommendation for reporting complications/adverse events (%)	46.5	59.1	26.8	45.7	53.3	39.3	35.4	52.3	43.4	37.9
Recommends EQUATOR Network listed reporting guidelines (%)	24.7	29.5	19.5	29.3	21	22.4	14.9	36	27.7	21
Surgery-specific RGs										
SCARE	24.8	29.8	19.5	29.3	21	22.4	14.9	36	27.7	21
STROCSS	24.7	29.5	19.5	29.3	21	22.4	14.9	36	27.7	21
PROCESS	24.8	29.8	19.5	29.3	21	22.4	14.9	36	27.7	21
Nonsurgery specific RGs										
CARE	28.1	33.7	22	34.2	26.7	23.4	17	39.6	30.1	24.2
CONSORT	41.7	50.5	19.5	43.9	50.5	36.5	29.9	51.4	42.2	34.7
SPIRIT	25.7	29.8	22	30.5	21.9	22.4	15.3	38.7	30.1	23.4
TREND	26.3	31.6	22	30.5	24.8	23.4	15.3	38.7	30.1	21.8
COMET (%)	0.1	0.3	0	0	0	0	0	0	0	0
ISPOR (%)	0.5	0.5	0	0	0	0.9	0	0.9	0	2.4
Other recommendations for reporting complications/adverse events (%)	8.4	12.5	2.4	3	27.7	5.6	8.5	0.9	2.4	0.8
Guidelines for reporting on vascular surgery	0.1	0.3	0	0	0	0	0	0	0	0
Guidelines for Reporting Total Ankle Arthroplasty (TAA) Problems and Complications Resulting in Re-Operation	0.1	0	0	0	0	0	0.4	0	0	0
Minimum Information for Studies Evaluating Biologics in Orthopaedics (MIBO)	0.1	0	0	0	0	0	0.4	0	0	0
Reporting and Grading of Complications After Urologic Surgical Procedures: An ad hoc EAU Guidelines Panel Assessment and Recommendations	0.4	0	0	0	4.8	0	0	0	0	0
Reporting Standards: Completeness and the Use of Reporting Guidelines	0.1	0	0	0	1.9	0	0	0	0	0
The American College of Cardiology Foundation/American Heart Association Task Force on Practice Guidelines For Reviews that synthesize findings from numerous studies into a single summary recommendation	0.1	0.3	0	0	0	0	0	0	0	0
Royal Australian and New Zealand College of Ophthalmologists Recommendations	0.1	0	0	0	0	0	0	0	0	0.8
Specific recommendation for reporting ‘complications’ (by journal)	6.3	11.1	2.4	3	11.4	5.6	6.6	0.9	2.4	0
Specific recommendation for reporting ‘intraoperative complications’ (by journal)	0.5	0.3	0	0	4.8	0	0.4	0	0	0
Specific recommendation for reporting ‘postoperative complications’ (by journal)	0.6	0.5	0	0	4.8	0	0.7	0	0	0

Percentages indicate relative endorsement of specific adverse event reporting recommendations/requirements. All results are reported as a percent of journals within the category.

CARE, CAse REport; CONSORT, Consolidated Standards of Reporting Trials; COMET, Core Outcome Measures in Effectiveness Trials; EAU, European Association of Urology; ENT, ear, nose, and throat; EQUATOR, Enhancing the QUAlity and Transparency Of health Research; ISPOR, International Society for Pharmacoeconomics and Outcomes Research; OB-GYN, obstetrics and gynecology; PROCESS, Preferred Reporting Of CasE Series in Surgery; RGs, Reporting Guidelines; SCARE, Surgical CAse REport; SPIRIT, Standard Protocol Items: Recommendations for Interventional Trials; STROCSS, strengthening the reporting of cohort, cross-sectional and case–control studies in surgery; TRENDS, Transparent Reporting of Evaluations with Nonrandomized Designs.

The multivariable logistic regression model revealed that, compared to journals in the first quartile (most influential), lower quartile journals had a lower likelihood of any AE reporting guidance (Table 3). By region, journals in North America, Western Europe, the Middle East, and Asiatic Regions had a comparable likelihood of AE reporting recommendations. In contrast, journals in Eastern Europe were less likely (OR: 0.19; 95% CI: 0.09–0.44; *P*<0.0001) to recommend surgical AE reporting. Surgical AE reporting was most commonly recommended/required by journals in urology, anesthesia, and general surgery.

**Table 3:** Multivariable logistic regression model of journal adverse event reporting recommendation by SJR quartile, region, and category.

	OR	95% CI low	95% CI high	*P*
SJR quartile				
Q1	Ref			
Q2	0.70	0.52	0.96	0.0243
Q3	0.40	0.29	0.55	<0.0001
Q4	0.25	0.17	0.35	<0.0001
Region				
Western Europe	Ref			
Northern America	0.79	0.61	1.04	0.0893
Asiatic Region	1.10	0.74	1.64	0.6228
Eastern Europe	0.19	0.09	0.44	<0.0001
Middle East	1.48	0.83	2.63	0.1795
Others	1.06	0.63	1.79	0.8252
Category				
General surgery	Ref			
Transplant	0.23	0.10	0.49	0.0002
OB-GYN	0.57	0.39	0.84	0.0047
Urology	0.81	0.51	1.29	0.3765
Otorhinolaryngology	0.44	0.28	0.69	0.0004
Orthopedics	0.40	0.29	0.56	<0.0001
Anesthesia	0.69	0.44	1.08	0.1022
Emergency medicine	0.50	0.30	0.84	0.0081
Ophthalmology	0.43	0.28	0.67	0.0002

OR indicates odds ratio; Q, quartile; SJR, SCImago journal rank.

## Discussion

In our study, we found that slightly less than 50% of journals recommended any form of AE reporting, and only one-fourth of those journals recommended EQUATOR network guidelines, more often in higher-tier journals. Reporting surgical AEs plays a vital role in academic surgical centers worldwide, many of which routinely hold morbidity and mortality conferences specifically dedicated to this subject. AE rates are often underreported, and approximately half of the reported AEs result from provider error, affording opportunities to improve the quality of care^19^. Such variations in reporting introduce potential biases, making the true incidence of AEs unclear.

In addition to evaluating generic recommendations within author guidelines regarding AE reporting, the authors of this paper have specifically chosen to evaluate reporting guidelines listed by the EQUATOR Network. The EQUATOR Network provides the most comprehensive, easily navigable, and openly accessible list of guidelines, yet only a fraction of journals specifically alluded to these standards. The key point is that there is general AE reporting guidance [e.g. Consolidated Standards of Reporting Trials (CONSORT)/CAse REport (CARE) type guidance], where regardless of the type of intervention. For researchers who aim to ensure that their publications meet the highest standards, the authors of this study recommend exploring the guidelines endorsed by the EQUATOR Network as a well-grounded first step. To cast a wide net regarding AE reporting, the authors included all guidelines which included ‘adverse events,’ or equivalent, within the checklist. Two such guidelines, which were referenced with the highest frequency among the included journals, were the CONSORT and CARE guidelines, which were referenced either directly or indirectly by 41.7 and 28.1% of journals, respectively^20^. Within the CONSORT guidelines, the CONSORT authors recommend reporting ‘All important harms or unintended effects in each group’ when reporting randomized trials^21^. Similarly, one of the follow-up items within the CARE guidelines is reporting ‘Adverse and unanticipated events’ when writing case reports. These examples illustrate the range of the verbiage captured in this analysis. To cast a wide net regarding AE reporting, the present study included all guidelines that referred to ‘adverse events,’ or equivalent, within the checklist. In addition to evaluating generic recommendations within author guidelines regarding complication reporting, the authors of this paper have specifically chosen to evaluate reporting guidelines listed by the EQUATOR Network. The EQUATOR Network provides the most comprehensive, easily navigable, and openly accessible list of guidelines. For researchers who aim to ensure that their publications meet the highest standards, the authors of this study recommend exploring the guidelines endorsed by the EQUATOR Network as a well-grounded first step. Of note, there is general AE reporting guidance (e.g. CONSORT/CARE type guidance), where regardless of the type of intervention AEs should be reported, and then there is surgery-specific AE reporting guidance.

Only 0.5% of journals had specific recommendations for reporting intraoperative AEs (iAE), possibly leading to a paucity of iAE reporting in clinical trials as described previously^8^,^22^. While a handful of iAE grading systems are available^23^–^28^, there are no common-shared guidelines regarding best practices in iAE reporting within the literature regarding perioperative outcomes. The preliminary iAE reporting guidelines and checklist developed by the ICARUS Global Surgical Collaboration group have been recently published^29^ and are currently undergoing global, multispecialty face validation^30^. Of course, it is also essential to acknowledge that publication-specific guidelines may indirectly impact patient outcomes. As described by authors involved in the World Health Organizations Surgery Saves Lives Program^31^ and comparable studies evaluating the utilization of structured debriefing, AE-related checklists improve organizational mindfulness and aid in achieving ideals of high-reliability organizations. and are undergoing global, multispecialty validation^30^. Publication-specific guidelines may indirectly impact patient outcomes. As described by authors of the World Health Organizations Surgery Saves Lives Program^31^ and studies evaluating structured debriefing, AE-related checklists improve organizational mindfulness and aid in achieving ideals of high-reliability organizations. and are currently undergoing global, multispecialty face validation^29^. Of course, it is also important to acknowledge that publication-specific guidelines may indirectly impact patient outcomes. As described by authors involved in the World Health Organizations Surgery Saves Lives Program^31^ and comparable studies evaluating the utilization of structured debriefing, AE-related checklists improve organizational mindfulness and aid in achieving the ideals of high-reliability organizations.

Intraoperative AEs are underreported compared to their postoperative AEs^10^,^14^,^22^,^29^,^30^,^32^. Our findings underscore the importance of the ICARUS (Intraoperative Complication Assessment and Reporting with Universal Standards)^10^,^29^,^32^–^34^ Global Surgical Collaboration Project. As of March 2022, the ongoing ICARUS survey had over 5000 responses from surgeons and anesthesia providers, of which over 90% agreed that it is crucial for academic journals to offer guideline recommendations for properly assessing, reporting, and grading intraoperative AEs^30^. Further, these providers felt that criteria checklists would be helpful adjuncts for properly assessing, grading, and reporting these events in scientific publications. Despite the findings by the ICARUS group regarding the importance of intraoperative AE capture, as of the time of the present study, only 46.5% of journals offer any type of AE reporting recommendation. These findings could be used to make a call to action about the relevance of reporting and measuring surgical AEs. It is obviously an essential tool for risk management, quality control, continuous quality improvement, and open ‘error culture’. Such global cross-specialties initiatives can help to increase awareness and could provide guidance on how to report surgical and anesthesiologic AEs related to interventions.

Surgical AEs and complications are reported and discussed at academic surgical centers worldwide, many of which routinely hold morbidity and mortality conferences specifically dedicated to this subject. Academic surgical centers worldwide, many of which routinely hold morbidity and mortality conferences specifically dedicated to this subject. A recent study found that complication rates were underreported, and approximately half of the reported complications resulted from provider error, which is a target area of improvement to enhance the quality of care^19^. These variations in complication reporting introduce potential biases in reporting clinical outcomes; therefore, due to these inconsistencies in reporting and underreporting, the data reporting on complication incidence is unclear.

Surgical AEs and complications are estimated to occur at rates two to four times higher than those reported by the Institute of Medicine, and approximately half of such events are avoidable incidents^19^. Despite this, inconsistent complication reporting is frequently discussed across surgical literature^7^,^11^,^27^,^35^. In addition, while standardized reporting guidelines are available for postoperative complications^4^, an equivalent does not yet exist for best practices in publishing *intraoperative* complications. As a caveat, this change would require prospective data collection, which would lead to a higher rate of complications than retrospective data acquisition.

There are several limitations to the present study. Namely, this study does not evaluate the level of recommendation by journals for AE reporting (i.e. there is no differentiation between requirements, endorsements, or suggestions). The authors intentionally approached the journal publication guidelines with a broad lens. Another limitation is that this study is one of the first to analyze the variation in these recommendations. Even if proper training was provided and a senior author supervised the data collection, this process might have a certain degree of intercollector disagreement. There is limited evidence in contemporary literature for a comparison of the present findings.

The unique findings of this study are the greatest strength. Given that journal recommendations were analyzed by quartile, region, and surgical category, the findings may help inform where we, as a scientific community, should focus our efforts. For example, while many Q1 journals may be appropriate for evaluating the impact of journal guidelines, recommendations, and requirements, there is much work to be done to improve the quality of research publications across the board. The authors of this study encourage journal editors and reviewers to select and endorse reporting guidelines for their respective audiences. Ensuring the highest quality evidence in all publications is the bedrock of future scientific progress.

The availability of AEs reporting guidelines has not improved since this issue first garnered attention. One reason change has yet to occur is that editorial boards may believe their current policies for reporting are sufficient, and they may be concerned that stricter reporting guidelines will deter researchers from submitting to their journals in favor of those with fewer guidelines. Another problem is that when journals take on these guidelines, the uptake is varied. Some journals are much more specific and attentive to these guidelines, and the level of endorsement varies as well^36^–^38^. Therefore, due to these barriers, it is imperative to understand the requirements and recommendations put forth by individual journals.

Looking forward, there are a variety of strategies that can be employed to promote guideline utilization in the medical literature. The EQUATOR Network published an instructional guide for journal editors describing best practices for introducing, choosing, and utilizing reporting guidelines^19^. Though, as has been discussed in the past, it is essential to consider the recommendation and the enforcement of such recommendations^39^. Journal endorsement is a practical first step. However, an endorsement is equivalent to a suggestion and not a requirement. Ultimately, if journals wish to ensure the highest quality publications, it may be valuable to consider requiring the submission of relevant checklists alongside manuscripts – a practice that has previously been documented with successful outcomes^40^.

## Conclusion

AE reporting guideline recommendation rates are inconsistent and often absent, with variation by journal quartile, subspecialty category, and region. Standardization of guidelines is a potential strategy to improve the quality of reporting and measurement of patient outcomes. Ultimately, grading and sharing AEs is of utmost importance in identifying, addressing, and preventing events associated with perioperative and postoperative morbidity and mortality.

## What is New?

- Less than half of surgery and anesthesiology journals require/recommend any form of adverse event (AE) reporting, with less than 1% of these journals specifically recommending intraoperative AE reporting.
- General surgery, urology, and anesthesiology journal categories are most likely to recommend AE reporting.
- The known issues with AE reporting likely reflect the lack of standardized reporting guidelines from journals.
- Journal editorial board endorsement of intraoperative and postoperative AE reporting is an essential first step to studying these events and their impact on patient morbidity and mortality.

## Ethical approval

Not applicable.

## Sources of funding

This research did not receive any specific grant from funding agencies in the public, commercial, or not-for-profit sectors.

## Author contribution

T.N.S., J.B., G.E.C.: study design, data analysis, and manuscript writing; A.S.S., A.L.R., and L.C.P.: study design and data collection; G.E.C.: supervision. All authors contributed and reviewed the manuscript.

## Conflicts of interest disclosure

Julie A. Sosa is a member of the Data Monitoring Committee of the Medullary Thyroid Cancer Consortium Registry, supported by GlaxoSmithKline, Novo Nordisk, Astra Zeneca, and Eli Lilly; Institutional research funding is received from Exelixis and Eli Lilly. No additional disclosures to report

## Research registration unique identifying number (UIN)

1. Name of the registry: not applicable.
2. Unique identifying number or registration ID: not applicable.
3. Hyperlink to your specific registration (must be publicly accessible and will be checked): not applicable.

## Guarantor

Giovanni E. Cacciamani.

## Supplementary Material
