# Supplementary material for: Bibliometric analysis of academic journal recommendations and requirements for surgical and anesthesiologic adverse events reporting
Source: Int J Surg. 2023 May 3;109(5):1489–96. doi: 10.1097/JS9.0000000000000323 (PMC10389352; doi:10.1097/JS9.0000000000000323)
Supplement: Supplementary file 1 [file js9-109-1489-s001.docx]

APPENDIX

Bibliometric Analysis of Academic Journal Recommendations And Requirements For Surgical And Anesthesiologic Adverse Event Reporting

Table A.1

|  | **Recommending complication reporting** | | **Non-Recommending complication reporting** | | *Student’s t-test  p-*value |
| --- | --- | --- | --- | --- | --- |
| **H index, median (IQR)** | 35 | (17-72.25) | 23 | (8-54.25) | <.0001 |
| **Total Docs. (2020), median (IQR)** | 113 | (0-276.25) | 77 | (34-149.25) | <.0001 |
| **Total Docs. (3years), median (IQR)** | 279.5 | (148.75-622.25) | 196.5 | (96.75-368) | <.0001 |
| **Total Refs., median (IQR)** | 3141 | (1479-6846.75) | 1916 | (793.75-4085.25) | <.0001 |
| **Total Cites (3years), median (IQR)** | 439 | (152.75-1346) | 176 | (33-608.5) | <.0001 |
| **Citable Docs. (3years), median (IQR)** | 241.5 | (132-502.75) | 168 | (86.75-327.25) | <.0001 |
| **Cites / Doc. (2years), median (IQR)** |  |  |  |  |  |
| **Publisher** |  |  |  |  | N/A |
| Elsevier | 102 | (52.6%) | 92 | (47.4%) |  |
| Springer | 83 | (66.9%) | 41 | (33.1%) |  |
| Lippincott Williams and Wilkins | 47 | (47.0%) | 53 | (53.0%) |  |
| Saunders | 34 | (58.6%) | 24 | (41.4%) |  |
| Wiley-Blackwell | 35 | (64.8%) | 19 | (35.2%) |  |
| BioMed Central | 17 | (47.2%) | 19 | (52.8%) |  |
| Taylor and Francis | 6 | (16.7%) | 30 | (83.3%) |  |
| SAGE | 43 | (82.7%) | 9 | (17.3%) |  |
| Wolters Kluwer | 23 | (62.2%) | 14 | (37.8%) |  |
| S Karger | 16 | (76.2%) | 5 | (23.8%) |  |

Table A.2

| Guidelines |
| --- |
| CARE |
| COMET |
| Common terminology criteria (CTCAE) |
| CONSORT |
| CTCAE (Common Terminology Criteria Adverse Event) |
| Guidelines for reporting on vascular surgery |
| Guidelines for Reporting Total Ankle Arthroplasty (TAA) Problems and Complications Resulting in Re-Operation |
| IPM_QRBNR |
| ISPOR |
| Minimum Information for Studies Evaluating Biologics in Orthopaedics (MIBO) |
| PROCESS |
| RANZCO |
| Reporting and Grading of Complications After Urologic Surgical Procedures: An ad hoc EAU Guidelines Panel Assessment and Recommendations |
| Reporting Standards: Completeness and the Use of Reporting Guidelines. |
| SCARE |
| SPIRIT |
| STROCSS |
| the American College of Cardiology Foundation/American Heart Association Task Force on Practice Guidelines for Reviews that synthesize findings from numerous studies into a single summary recommendation, |
| TREND |

Table A.3a

| **Post-operative Adverse Event Report Examples** |
| --- |
| Total Ankle Arthroplasty (TAA) Articles  TAA manuscripts must have a minimum of two-year follow-up for all patients in the study. Complications or implant issues can be reported with shorter follow-up. All repeat surgeries must be documented for all time points from the time of the index TAA The template below should be used as a guide for authors in reporting complications resulting in reoperation(s). This is based on the COFAS Ankle Arthritis Study Group coding system for reporting reoperations. Overlap will occur for some of these categories as individual patients may have had more than one re-operation. The intent is to distinguish between different types of complications resulting in reoperation (instead of simply reporting that a certain number of patients in a study had re-operations). The Editorial Board of FAI also recognizes the need for an international consensus in reporting TAA outcomes. This includes patient reported outcomes, objective clinical and radiographic measurements, and a system for reporting complications and adverse events. TAA joint registries are also essential so that we can learn from our successes but also from our failures and maintain surveillance to identify non-random causes for failure. Repeat surgery can be reported individually or as a series of events. The authors should outline which method was used. Documentation of resource utilization (additional surgery time, days in hospital or additional clinic visits) for repeat surgery will assist in determining the impact of the complication or repeat surgery. Non-surgical complications (such as DVT, pulmonary embolism or infection treated by antibiotics) should also be documented. 13.1 Guidelines for Reporting TAA Problems and Complications Resulting in Re-Operation  No reoperation Hardware removal related to TAA  (e.g. medial malleolus screw(s), fibular plate) Subsequent operative treatment related to TAA but not involving TAA components  (e.g. osteotomy, fusion of other joint(s) of the foot, ligament repair/reconstruction) Operative treatment of periprosthetic fracture(s)  a. At the time of the index procedure  b. After the index procedure Debridement of gutter or heterotopic ossification with retention of components Polyethylene liner exchange for polyethylene wear or fracture Operative treatment of postoperative arthrofibrosis  (e.g. joint debridement +/- capsulotomy, Achilles lengthening, PE liner exchange), Debridement +/- grafting of osteolytic cyst(s)  (with retention of metal components +/- polyethylene exchange) Operative treatment of superficial infection or incision problem  (e.g. I & D, secondary closure) Operative treatment of deep infection requiring debridement   (+/- polyethylene liner exchange but NOT removal of metal components) Deep Infection requiring removal of ALL components   a. One stage TAA revision to TAA  b. Two stage TAA revision to TAA  c. Conversion to “long term” cement spacer  d. Conversion to arthrodesis Revision of metal components because of implant failure  (e.g. aseptic loosening, subsidence, malposition, implant fracture----not for infection)  a. Tibia  b. Talus  c. Both tibia and talus revised to TAA  d. Removal of components and conversion to arthrodesis Amputation related to complications from TAA  State reason(s): pain, infection, vascular compromise, failed prior treatments, patient choice, etc. |
| Complication  Complications are an inherent aspect of Operative Neurosurgery. These articles should consist of a description of a surgical (intraoperative or postoperative) complication that is unique in its occurrence or causation. It should include a concise clinical summary, images (diagnostic and intraoperative as appropriate) to illustrate the pertinent issues, and a scholarly discussion of the root cause analysis and how it could have been prevented in the reported case and similar cases that other surgeons and readers may encounter. |
| Reporting and Grading of Complications After Urologic Surgical Procedures: An ad hoc EAU Guidelines Panel Assessment and Recommendations |
| Clavien-Dindo classification of surgical complications and EAU guidelines panel assessment and recommendations |
| Guidelines for Reporting Total Ankle Arthroplasty (TAA) Problems and Complications Resulting in Re-Operation |
| Category "Surgical Techniques" Author guidelines |

Table A.3b

| **Intraoperative Adverse Event Report Examples** |
| --- |
| Complications are an inherent aspect of Operative Neurosurgery. These articles should consist of a description of a surgical (intraoperative or postoperative) complication that is unique in its occurrence or causation. It should include a concise clinical summary, images (diagnostic and intraoperative as appropriate) to illustrate the pertinent issues, and a scholarly discussion of the root cause analysis and how it could have been prevented in the reported case and similar cases that other surgeons and readers may encounter. A structured abstract of no more than 250 words is required. Maximum length: 2,125 words of text (not including abstract, references, figures, tables, and online-only material). |
| Reporting and Grading of Complications After Urologic Surgical Procedures: An ad hoc EAU Guidelines Panel Assessment and Recommendations |
| EAU guidelines panel assessment and recommendations |
| Category "Surgical Techniques" Author guidelines |
